# A rare *Fusarium equiseti* infection in a 53-year-old male with burn injury: A case report

**DOI:** 10.18502/cmm.7.1.6245

**Published:** 2021-03

**Authors:** Tang Xuan Hai, Nguyen Thai Ngoc Minh, Do Ngoc Anh, Tran Ngoc Dung, Ngo Thi Minh Chau, Le Tran-Anh

**Affiliations:** 1 Department of Otorhinolaryngology, Nghe An Obstetrics and Pediatrics Hospital, Vinh, Vietnam; 2 Intensive Care Unit, National Hospital of Burn, Vietnam Military Medical University, Hanoi, Vietnam; 3 Department of Parasitology, Vietnam Military Medical University, Hanoi, Vietnam; 4 Department of Pathology, Hospital 103, Vietnam Military Medical University, Hanoi, Vietnam; 5 Department of Parasitology, Hue University of Medicine and Pharmacy, Hue, Vietnam

**Keywords:** Burns, Fungal wound infections, Fusariosis, Fusarium equiseti, Vietnam

## Abstract

**Background and Purpose:**

Burn injuries are prone to infection caused by bacteria, fungi, or other pathogens. Fungal wound infection usually has non-specific clinical symptoms. Nevertheless, in some cases, the fungal burden is so substantial that can easily be seen by the naked eyes, but this phenomenon has rarely been reported with *Fusarium*.

**Case report:**

A 53-year-old patient with severe burn injury was admitted to the intensive care unit of the National Hospital of Burn, Ha Noi, Vietnam. His wound was dressed with a traditional herbal product before the hospital admission. On the 5th day after the admission, some white patches suspected of fungal colonies appeared on burn lesions where the herbal medicine was placed. Histological examination (Periodic acid-Schiff) and culture of biopsy samples taken from those lesions revealed fungus that was identified as *Fusarium equiseti* after analysis of the internal transcribed spacer and D1/D2 region of the large subunit of the 28S rDNA. The isolated strain showed susceptibility to voriconazole but resistance to fluconazole, itraconazole, caspofungin, and amphotericin B *in vitro*. The patient received aggressive treatment, including IV voriconazole (400 mg daily from day five); however, he could not recover.

**Conclusion:**

*Fusarium* should be suspected in burn patients with white patches on lesions. Antifungal susceptibility testing is important since multidrug resistance is common among *Fusarium* strains.

## Introduction

Burn injuries are among the most common and deleterious types of all injuries. Burn wound disrupts the normal barrier function of the skin and incites significant immune dysfunction; therefore, burn patients are prone to infection which relates to significant mortality and morbidity [ 1 ]. The most common causative agent of infection in burn patients is bacteria followed by fungi or viruses [ 1 ]. The source of fungal infection is either endogenous (from the commensal ﬂora of burn patients), or exogenous (from the hands of health care personnel [ 1 ], surrounding environment [ 2 ], or materials used for burn management) [ 3 ]. 

Invasive fungal infections in burn patients include localized (fungal wound infection [FWI]) or disseminated (fungemia) disease [ 1 ]. Patients with FWI usually have non-specific clinical symptoms but in some cases, the causative agent is highly suspected when the fungal burden is so substantial as to be easily seen by the naked eye-a phenomenon that has been reported with *Aspergillus* [ 4 ], *Absidia* [ 5 ] but not with *Fusarium*. 

Vietnam is a developing country; this means that Vietnamese people are considered at a higher risk of burns, compared to those in developed countries [ 6 ]. In Vietnamese traditional medicine, herbal sources still play an important role in the treatment of different diseases, including burn injuries [ 7 ]. However, these traditional medications are potentially contaminated with pathogens that are the source of local infection when used in topical applications [ 8 ]. It seems likely that there is a proportion of burn patients infected with pathogens from traditional products but no definite evidence has been published. This report presents a patient with the colony appearance of *Fusarium* on burn lesions that were dressed with a traditional medication in Vietnam. 

## Case report

A 53-year-old male was extensively burnt after falling into a boiling water tank. The patient was pulled out from the tank and transferred to a private medical facility where his wound was immediately dressed with a traditional herbal product. Two h after the accident, the patient was admitted to the intensive care unit (ICU) of the National Hospital of Burns, Hanoi, Vietnam on 18th June 2020 with severe shock. 

Physical examination revealed extensive injuries involving 95% (with a deep injury proportion of 56%) of the total body surface, including his face, chest, abdomen, back, and all extremities. Moreover, a yellow-brown layer of the herbal medications adhered to the surface of the blisters. The patient was managed according to standard care for severely burned patients, such as aggressive resuscitation, surgical excision, and empirical antibiotic therapy. 

**Figure 1:**
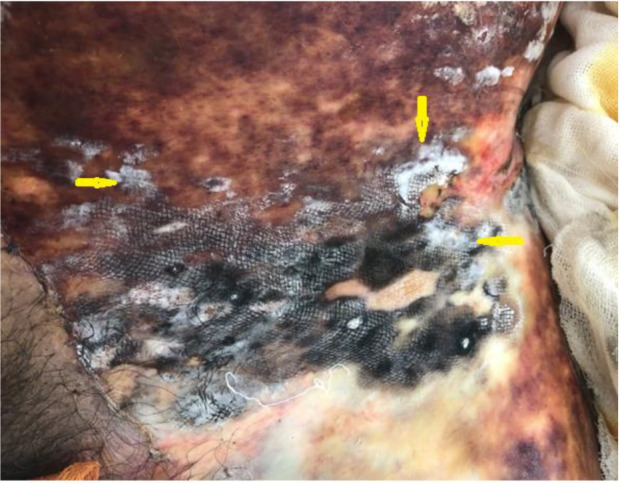
White patches that were suspected of fungal infection on the surface of burn lesions (yellow arrows)

His wound was cleaned and dressed with covering materials and topical antimicrobial agents, including chlorhexidine, povidon-iod, and nanosilver ointment. On day five, some white patches suspected of fungal colonies appeared on burn lesions where the herbal medicine was placed (Figure 1) and intravenous voriconazole (400 mg daily) was added to the treatment.

**Figure 2:**
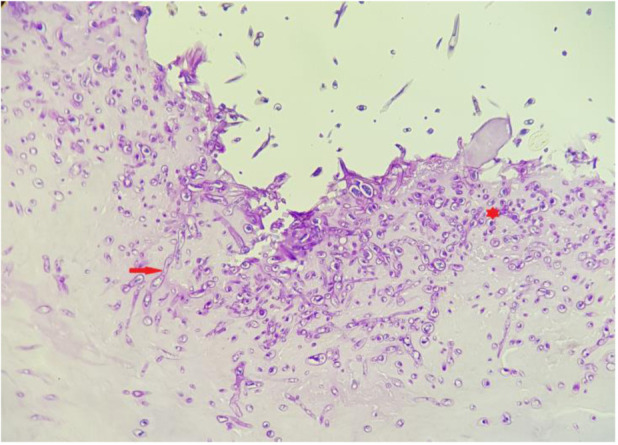
Varicosities hyphae (arrow) and yeast-like structures (star) of *Fusarium* on the slide stained with Periodic acid-Schiff (day 6).

Despite the active treatment, the general condition of the patient deteriorated with septic shock and multiple organ failures. Wound swab culture grew Acinetobacter baumannii, Staphylococcus aureus (on Blood agar, Merck, Germany), and *Fusarium* (on Sabouraud Dextrose Agar, Bio-Rad, France). On day six, a biopsy was taken from the lesion covered by the white patches and transferred to the laboratory. Histological examination (Periodic acid Schiff) showed abundant varicosities hyphae and yeast-like structures suggesting *Fusarium* infection in the tissue and on the base of the ulcer (Figure 2).

**Figure 3:**
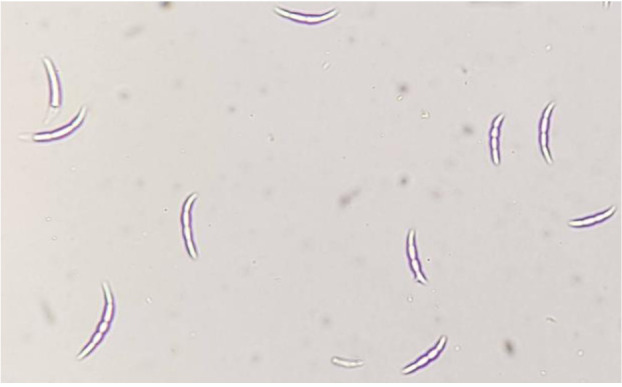
Crescent-shaped macroconidia of *Fusarium* under microscopic examination

The culture of biopsy material performed on Sabouraud dextrose agar grew a white colony with many crescent-shaped macroconidia characteristic of *Fusarium* under the microscope (Figure 3).

**Figure 4:**
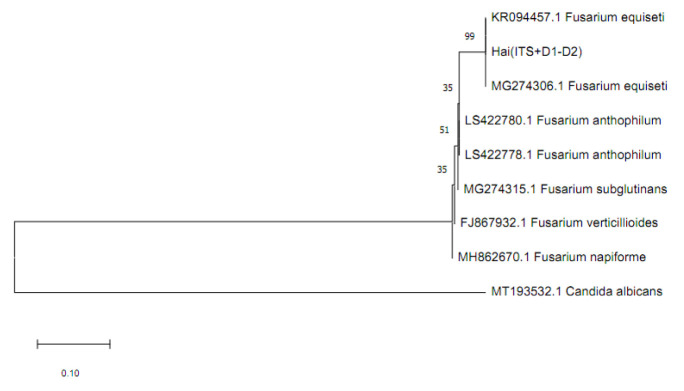
Neighbor-joining phylogenetic tree to identify fungal species

The DNA from the pure culture was extracted by Fungi/Yeast Genomic DNA Isolation Kit (Norgen BiotekCorp., Canada). The internal transcribed spacer (ITS) and D1/D2 region of the large subunit of the 28S rDNA was amplified and sequenced by using a pair of primers (forward ITS5 and reverse NL4). The polymerase chain reaction was run by 40 cycles at 95°C for 30 sec, at 55°C for 30 sec, at 72°C for 60 sec, and finally at 72°C for 10 min. The fungus was identified as *Fusarium equiseti* by GenBank BLAST and phylogenetic analysis using Bioedit software (version 7.0) and MEGA software (version 7) (Figure 4). The obtained sequence was submitted to GenBank [MW131978]).

Antifungal susceptibility testing by the agar disk diffusion procedure was performed according to Espinel-Ingroff et al. [ 9 ]. Briefly, the entire surfaces of the Mueller Hinton Agar (Hardy Diagnostics, Santa Maria, CA) plates (150mm) were inoculated with a suspension of the tested mold and then applied with disks containing antifungals. Inhibition zone diameters (ID) were measured at the point where there was a notable reduction of growth 72 h after incubating these plates in ambient air at 35°C. The isolation was resistant to fluconazole (ID, 13mm), itraconazole (ID, 12mm), caspofungin (no inhibition zone), and amphotericin B (ID, 11mm); however, it was susceptible to voriconazole (ID, 50mm). Due to the severe condition of the patient, his family asked for his discharge on day 10 and he died later.

### Ethical Form

Written informed consent was obtained from the legal guardian for publication of this case report and accompanying images. A copy of the written consent is available for review by the Editor-in-Chief of this journal on request.

## Discussion

*Fusarium* isolates are frequently isolated from natural environments and have about 70 species that have been reported to cause disease in humans (fusariosis) [ 10 ]. Humans can be infected with *Fusarium* through inhalation of airborne conidia or via breaks in the skin. In burn patients, *Fusarium* can cause FWI or fungemia [ 11 ]; it is the second most common agent of FWI due to molds, while infection with gross development of fungal colonies, like the present case, has rarely been reported [ 11 ]. 

The diagnosis of fusariosis, in this case, was suspected after discovering varicose hyphae and yeast-like structures on histopathological examination of biopsy specimens [ 12 ] and confirmed by the recovery of *Fusarium* with culture method [ 13 ]. The isolated fungus was identified as *F. equiseti*, a rare pathogen [ 10 ], using the molecular tool, analyzing both the ITS and D1/D2 region as recommended by some authors [ 14 ]. 

The resistance to many antifungals (i.e., fluconazole, itraconazole, amphotericin B, and caspofungin) of the isolated fungus was consistent with other reports [ 15 ]. Similar to other studies, the isolated fungus was susceptible to voriconazole [ 16 ] which appears to be more clinically effective and is used more frequently than amphotericin B for the treatment of fusariosis [ 17 ]. The patient was empirically treated with parenteral voriconazole once fusariosis was suspected as no topical antimicrobial agents are effective against invasive mold infections [ 18 ]. 

The outcome of the antifungal therapy in the present case is difficult to assess due to the severe underlying condition; however, local infection by *Fusarium* is associated with rather low mortality [ 11 ]. As the patient suffered from a severe burn injury and was infected with several pathogenic agents, the cause of the mortality is difficult to determine.

It is worth noting that there was a probable connection between the herbal medication and infection as the fungal colonies appeared on lesions dressed with the unknown herbal medicine. Vietnam is an Eastern country with developed traditional medicines and Vietnamese people continue to use many types of herbal medications for the treatment of different diseases, including burn injuries [ 7 ]. The efficacy and safety of these medications have scarcely been biologically and clinically validated with modern Western methods [ 19 ], and they are potentially contaminated with pathogens [ 8 ]. 

The association between herbal medicine and fungal infection, in this case, cannot be determined as there were no samples of the herbal medications to be tested. Nevertheless, in our opinion, the possibility of a connection should not be ruled out. *Fusarium* is present ubiquitously in natural environments and presumably proliferate on medicinal plants prepared in unhygienic conditions [ 20 ]. The possibility of a nosocomial infection cannot be excluded; however, fusariosis infection was not observed in other patients in the ICU.

## Conclusion

This study reported a rare case of fungal wound infection with the gross occurrence of *F. equiseti* on the surface of burn lesions. The possible association between herbal medication and infection demonstrates the potential risks of using unconventional medicines for burn management. Standard care and rigorous application of infection control measures are needed to prevent fungal infection for burn patients. Active surveillance and strict control measures in the preparation and usage of traditional medicines are needed. Besides, education to guide standard care for burn wounds is indispensable. Antifungal susceptibility testing is important since multidrug resistance is common among *Fusarium* strains.

## Authors’ contribution

T. X. H and L. T. A. developed the concept, designed the study, performed the literature search, and prepared the draft of the manuscript. N. T. N. M. collected the clinical data and samples. T. N. D. carried out histological examinations. D. N. A. and T. A. L. performed the culture and molecular analysis, N. T. M. C. performed the antifungal susceptibility test. 

## Financial disclosure

The authors received no financial support for the research, authorship, and/or publication of this article.
